# Soft-Sediment Deformation Structures Interpreted as Seismites in the Kolankaya Formation, Denizli Basin (SW Turkey)

**DOI:** 10.1155/2014/352654

**Published:** 2014-07-24

**Authors:** Savaş Topal, Mehmet Özkul

**Affiliations:** Department of Geological Engineering, Engineering Faculty, Pamukkale University, Kınıklı Campus, 20070 Denizli, Turkey

## Abstract

The NW-trending Denizli basin of the SW Turkey is one of the neotectonic grabens in the Aegean extensional province. It is bounded by normal faults on both southern and northern margins. The basin is filled by Neogene and Quaternary terrestrial deposits. Late Miocene- Late Pliocene aged Kolankaya formation crops out along the NW trending Karakova uplift in the Denizli basin. It is a typical fluviolacustrine succession that thickens and coarsens upward, comprising poorly consolidated sand, gravelly sand, siltstone and marl. Various soft-sediment deformation structures occur in the formation, especially in fine- to medium grained sands, silts and marls: load structures, flame structures, clastic dikes (sand and gravely-sand dike), disturbed layers, laminated convolute beds, slumps and synsedimentary faulting. The deformation mechanism and driving force for the soft-sediment deformation are related essentially to gravitational instability, dewatering, liquefaction-liquidization, and brittle deformation. Field data and the wide lateral extent of the structures as well as regional geological data show that most of the deformation is related to seismicity and the structures are interpreted as seismites. The existence of seismites in the Kolankaya Formation is evidence for continuing tectonic activity in the study area during the Neogene and is consistent with the occurrence of the paleoearthquakes of magnitude >5.

## 1. Introduction

Soft-sediment deformation structures are the result of liquefaction or fluidization in water-saturated unconsolidated sediments. Liquefaction or fluidization may be caused by various natural processes [1–4]. Soft-sediment deformation structures related to seismically induced liquefaction or fluidization are named as seismites [5]. Many researchers have studied seismites in different sedimentary environments [6–17]. In addition, there have also been experimental studies undertaken [2, 18, 19]. Seismites in lacustrine deposits are divided into different classes and their trigger mechanisms discussed by Sims [6], Alfaro et al. [11], Rodríguez-Pascua et al. [12], Bowman et al. [14], and Neuwerth et al. [16]. Sims [6] suggested that the relative abundance of seismites in lacustrine deposits is related to the following parameters: (1) the presence of water-saturated sediments, (2) the presence of sediments with high susceptibility to liquefaction, and (3) the absence of hydrodynamic and sedimentary processes obliterating the products of seismically induced deformation.

The aim of this paper is to describe the various types of soft-sediment deformation structures from the Kolankaya Formation in the Denizli Basin (western Turkey) and to discuss their potential triggering mechanisms.

## 2. Geological Setting

One of the most important neotectonic areas in Turkey is the horst and graben system of the Western Anatolia. Various geodynamic models have been proposed for neotectonic evolution of this graben: (1) tectonic escape model [20, 21], (2) back-arc extension model [22], and (3) orogenic collapse model [23].

In the recent years, a number of studies have also focused on the geodynamic setting, tectonic development, and stratigraphic of the Denizli Basin and its surroundings, in an attempt to understand the tectonic and palaeogeographic evolution of the Eastern Mediterranean and Aegean region [24–27].

The western Anatolian extensional province is characterized by basins trending NE-SW and E-W, earlier referred to as cross-grabens [21]. Their development and the causes and timing of crustal extension in southwestern Anatolia have been the subject of an ongoing debate [23, 28–30] and references therein. A general model for the extension of brittle upper crust in the Denizli region was proposed by Westaway [24]. He suggested that the Early-Middle Miocene reddish conglomerates of the lowermost part of the basin-fill succession were deposited prior to extension-driven tectonic subsidence, considered to have been initiated in the Denizli region not before the Late Miocene. More recently, Westaway et al. [31] and Kaymakçi [26] investigated the timing of the present phase of extension and kinematic development of the Denizli Basin and concluded that the Early-Middle Miocene alluvium was deposited in a poorly understood predecessor of the modern Denizli Basin. According to Westaway et al. [31], the present phase of crustal extension in the Denizli region began around 7 Myr BP. A model of pulsed extension was proposed for the western Anatolian grabens by Purvis and Robertson [32, 33]. In this model, major grabens were formed by a phase of Early to Late Miocene extension related to the roll-back of the Aegean subduction zone. These basins were later cut by grabens trending E-W, during a Pliocene phase of extension related to the westward “tectonic escape” of Anatolia. The model proposed that the E trending Alaşehir graben to the northwest of the Denizli Basin began to form in the Early Miocene and remained active until the recent. Koçyiğit [25] suggested that the Denizli Basin developed through two-stage extension, with one phase in the Middle Miocene-Middle Pliocene and the other in the Pliocene-Recent, and that these phases were separated by a phase of compression in the latest Middle Pliocene.

**Figure 1:**
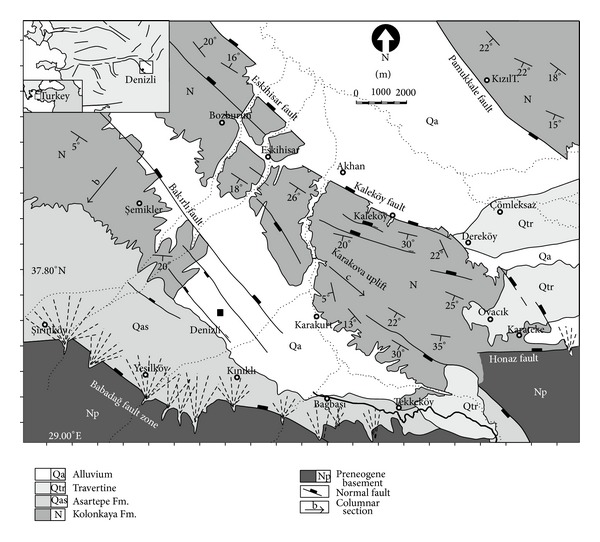
Generalization tectonic features and location study area (modified from [34, 56]).

The Denizli Basin NW-SE is a graben bounded by the Pamukkale fault to the north and the Babadağ fault to the south (Figure 1). The basin is 50 km long and 15–20 km wide and filled by Neogene-Quaternary continental deposits. The basement rocks are mapped as pre-Neogene units in this study (Figure 1). The Early-Middle Miocene Kızılburun Formation unconformably overlies this pre-Neogene basement rocks; the Middle Miocene Sazak Formation, and the Late Miocene-Late Pliocene Kolankaya Formation [27] are described in previous studies as the Denizli Group [34].

The Asartepe Formation with travertines and alluvium of Quaternary ages rest with angular unconformity on the Denizli Group. According to the sedimentological study performed at the western side of the basin, the basin originated as a half graben in the early Miocene and evolved into an asymmetric graben in the Quaternary [27].

The soft-sediment deformation structures investigated in this study occur in the Kolankaya Formation exposed along the NW-SE trended Karakova horst uplift (Figure 1). At the western part of the basin from base to the top, the Kolankaya Formation is represented by shallow lake-, deep lake- and fluviolacustrine deposits [27]. The formation is represented by only deep lake- and lacustrine fan delta deposits in the present study area. The formation is approximately 500 m thick and consists of clayey limestone, marl, unconsolidated sands, siltstone alternations and lenticular pebble horizons. Gray-beige-coloured marls are fine to medium-bedded and abundantly fossiliferous (Miocene:* Parapodiums *sp.,* Huerzelerimys/Castromys*,* Ictitherium robustum*,* Ictitherium *cf.* tauricum eximia*,* Machairodus aphanistus*,* Orycteropus *sp.,* Hipparion *sp.,* Ceratotherium neumayri, Chilotherium schlosseri, Dicoryphochoerus *sp.,* Samotherium boissieri, Palaeotragus *cf.* coelophrys, Gazella *cf.* capricornis, Gazella *aff.* gaudry, Oioceros wegneri, Choerolophodon pentelici. *Pliocene:* Mimomys pliocaeinicus, Borsodia sp.; MN17*). The sandy layers are usually poorly bedded, unconsolidated, and medium to coarse-grained and dark yellow to brown in colour.

## 3. Soft-Sediment Deformation Structures and Their Classification

Classification of the soft-sediment deformation structures is based on morphological features. In the present study, classifications and terms suggested by Lowe [35], Brenchley and Newall [36], Mills [37], Owen [1, 38], and Neuwerth et al. [16] were preferred and divided into areas studied in the three different groups ((1) load casts, drop and flame structures; (2) clastic dikes and sills; (3) disturbed laminitis, convolute laminations, slumps, simple recumbent folds, and synsedimentary faults) on the basis of their morphological features.

## 4. Soft-Sediment Deformations and Their Driving Forces in the Kolankaya Formation

**Figure 2:**
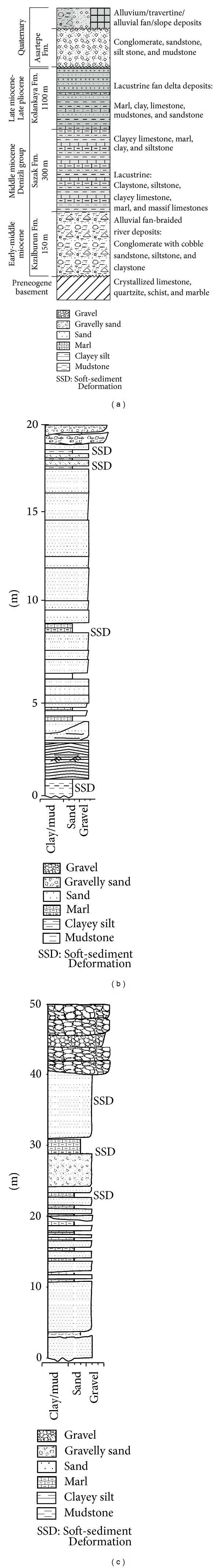
(a) Composite stratigraphy of the Denizli basin-fill succession (not to scale; after Alçiçek et al. [27]). (b) and (c) Representative columnar sections including soft-sediment deformation structures, measured from Kolankaya Formation exposed at the western part of the study area.

Soft-sediment deformation structures in the Kolankaya Formation are encountered mostly in the area of the Karakova uplift (Figure 1). The most frequently deformed lithologies are restricted to fine to medium-grained sands, marl, and gravely sand. The following structures have been observed (Figures 2(b) and 2(c)).

### 4.1. Load Casts, Drop, and Flame Structures

#### 4.1.1. Load Casts

**Figure 3:**
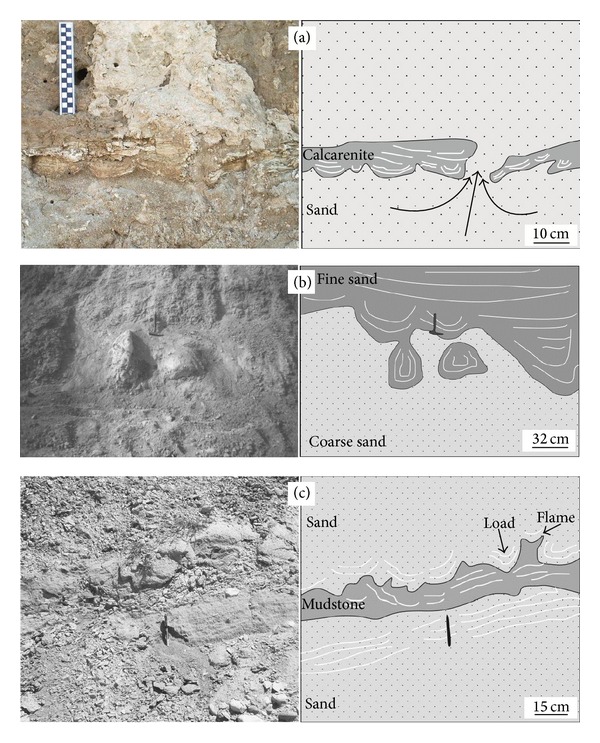
(a) Widely observed load structure seen in the study area and mainly formed in calcarenite and sands (37.770 N, 29.146 E). (b) This structure which is formed in fine and coarse-grained sand and named by Alfaro et al. [40] similar to drop structures. About 40 cm of material sunk into coarse-grained sand with detachment occurring in a later deformation phase (37.795 N, 29.154 E). (c) Load and flame structures observed in 15 cm thick mudstones in sand (37.773 N, 29.157 E).

Classification of these structures was made based on the criteria suggested by Owen [38]; they are the most common structure in the study area. The structures range in size from 1 to 40 cm and generally occur in calcarenite, sand, and silt (Figures 3(a) and 3(b)). They show slight penetration into the underlying material and a typical concave profile. The origin of the load casts in the Kolankaya formation is thought to be mostly related to a reverse density gradient [39]. The gravitational readjustment leads simultaneously to a descent of the denser sediment and an ascent of the lighter sediment. These types of structures are similar to the “sagging load cast” described by Alfaro et al. [40].

#### 4.1.2. Driving Force

The structures are formed in response to gravitational instability [19]. The resulting deformation depends upon the contrast of dynamic viscosities [11, 39]. The force required is linked to lateral variations in the distribution of sediment load when the substrate is liquidized and loses its capacity to support overlying sediment [38].

#### 4.1.3. Drop Structures

They are rare in the study area and developed in fine to coarse-grained sands. They typically average 60 cm in diameter (Figure 3(c)) and show features similar to those described by Anketell et al. [39] and Alfaro et al. [11]. They are formed by the sinking of load casts into water-saturated fine sediments, synonymous with the “pseudonodules” of Kuenen [18].

#### 4.1.4. Driving Force

Drop structures have a similar origin to load casts but are associated with a more advanced stage of deformation [11, 38–41]. According to Alfaro et al. [11], in some cases the structure seems unrelated to a difference in density. In this case, it is likely that the deformation is related to uneven loading, a similar mechanism to that postulated by Rodríguez-Pascua et al. [12] for the origin of some pseudonodules.

#### 4.1.5. Flame Structures

Flame structures are common in the study area and are generally formed in sands, muds, and marls. The structures range from 5 to 30 cm in size (Figures 3(c) and 6(b)). Poorly developed types of this structure are termed “mud diapirs” [38]. The structures always occur with load casts as seen in Figure 3(c). Consequently, the flame structure is developed by underlying mudstones which is injected into overlying sandstones.

#### 4.1.6. Driving Force

Flame structures owe their existence to large differences in dynamic viscosity between sediment layers [39] and are formed by fine-grained sediments behaving as diapiric intrusions [37].

### 4.2. Clastic Dikes and Sills

**Figure 4:**
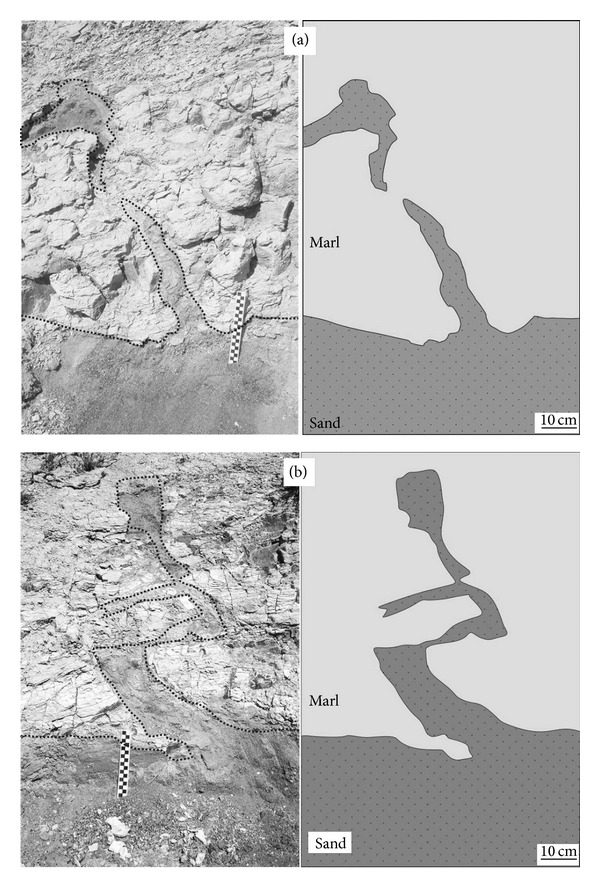
Clastic dikes that are seen in the study area. (a) In this figure coarse-grained sand clearly intrudes overlying layered marls (37.768 N, 29.152 E). (b) Sills and dikes of fine-grained sand in marls (37.768 N, 29.154 E).

**Figure 5:**
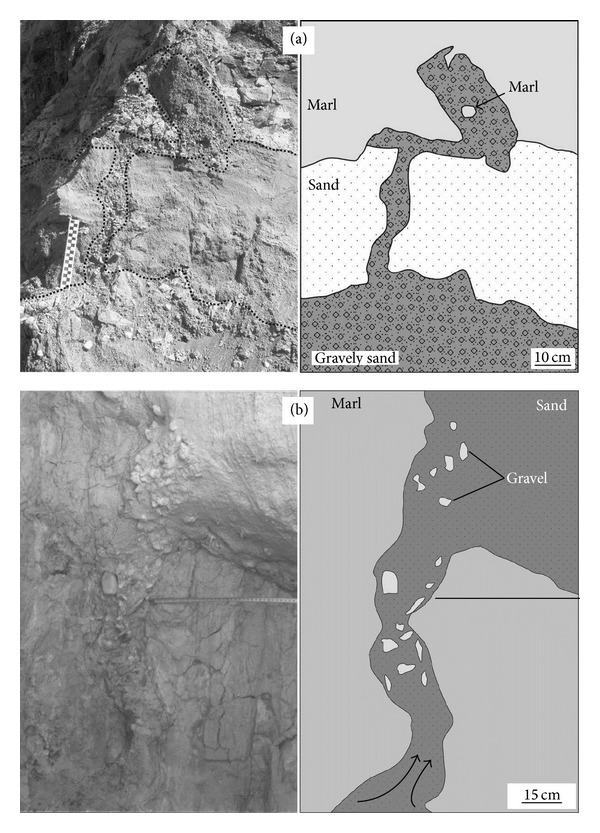
Clastic dikes in the study area. (a) Gravelly coarse-grained sand cuts through the sand layer and is intruded into overlying marls (37.769 N, 29.155 E). (b) Clastic material with fragments up to a max. 4 cm of gravel size intruded through marls (27.792 N, 29.130 E).

The dikes exposed in the study area are generally developed as coarse-grained and gravelly sands intruding marls. Gravel and marl fragments are observed in the intruded sands (Figures 5(a) and 5(b)). The patterns of dikes are variable and their sizes are typically 30 cm to 1 m (Figures 4(a), 4(b), 5(a), and 5(b)). Sills are emplaced parallel to the surfaces of layers without any internal structures and range in size between 20 and 30 cm. If there is no colour difference between sills and depositional layers this structure could be difficult to spot in the field.

Dikes are linked with a source-bed emplaced beneath, similar to the structures described by Rodríguez-Pascua et al. [12]. As a result of liquefaction, bending of the layer edges is characteristic feature of dikes. Dikes filled with sand containing some gravel and silt are very common. While mainly sand was vented, large quantities of vented gravel also occurred commonly [42]. According to Rodríguez-Pascua et al. [12], such examples are associated with the force of the trigger mechanism that caused the violent upward injection. Sills are associated with lateral sand flow which also causes upward/downward bending of layers. In both cases, deformation depends on liquefaction of the underlying sand source-beds.

#### 4.2.1. Driving Force

These structures are formed by intrusion of liquidized sands [12], interpreted as the result of liquefaction triggered by seismic shocks. The liquefaction is interpreted as resulting from water-saturated material with high pore water pressures moving upward [12, 43].

### 4.3. Disturbed Laminitis, Convolute Laminations, Slumps, and Synsedimentary Faults

#### 4.3.1. Disturbed Laminitis

**Figure 6:**
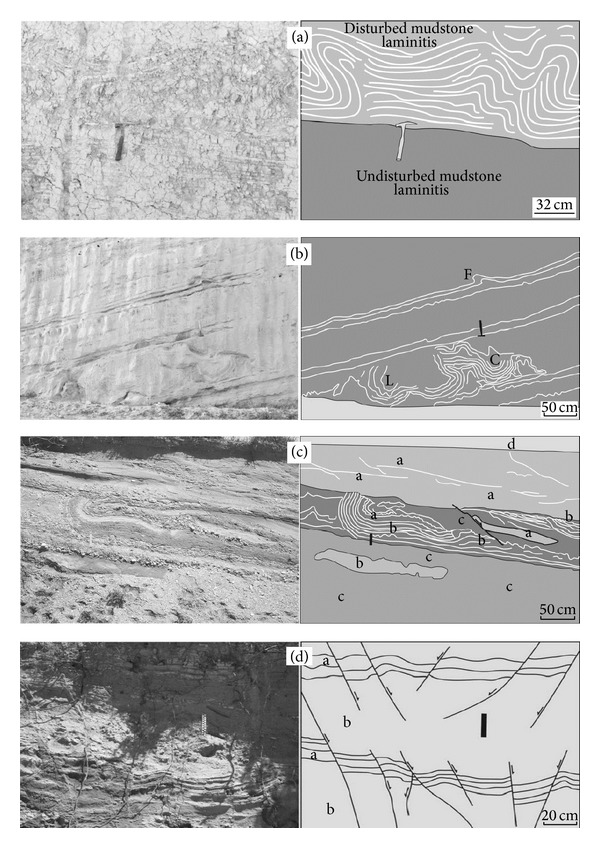
Other soft-sediment deformation structures: (a) disturbed laminitis (37.804 N, 29.132 E), (b) convolute laminations (F: Flame structures, L: Load casts, and C: Laminated convolute beds) (37.807 N, 29.115 E), (c) slump structure (a: coarse-grained sands, b: fine-grained sands, and c: gravelly unit) (37.765 N, 29.144 E), (d) synsedimentary faults (a: calcarenite, b: sand) (37.754 N, 29.178 E).

These structures are formed by weathering of mudstone layers. Weathering is observed approximately 70 cm thick at the top of horizontally spread mudstone layers (Figure 6(a)).

#### 4.3.2. Driving Force

The same structure was defined by Rodríguez-Pascua et al. [12] and interpreted as product of ductile deformation. Although there is no change in the thickness of layers, flexural bending observed and interpreted as forming due to resistance against ductile deformation [44].

#### 4.3.3. Convolute Laminations

The structures are observed in medium to coarse-grained sands but are rarely encountered in the study area. It is associated with load and flame structures (Figure 6(b)). It develops in part of a 1 meter thick unit in the succession. The shape of the structure appears to be defining anticlinal and synclinal patterns (Figure 6(b)).

#### 4.3.4. Driving Force

Convolute laminations are suggested to represent complex load structure although there is controversy about the origin of such structures [35, 37, 45]. We relate them to hydroplastic deformation and soft-sediment intrusion as suggested by Plaziat and Ahmamou [46].

#### 4.3.5. Slumps

This deformation structure occurs in fine-grained sand to gravel and is common in the study area (Figure 6(c)). Thickness of slumps varies between 90 cm and 130 cm; their shapes can clearly be seen to be folds. Axes of these folds are horizontal or nearly horizontal. The structure is seen associated with load structures, convolute laminations, and synsedimentary faults (Figure 6(c)).

#### 4.3.6. Driving Force

The structures movement of under consolidated sediments under the influence of gravity according to Moretti and Neuwerth et al. [13, 16]. The failure occurs responsible for the slump when the sediments are steepened beyond the stable angle of repose [37].

#### 4.3.7. Synsedimentary Faults

Closely spaced synsedimentary faults displace alternating coarse-grained sand beds and gray-coloured laminated marl over intervals c. 1 m (Figure 6(d)). The faults illustrated in the Figure 6(d) are normal structures with offsets between 5 and 15 cm.

#### 4.3.8. Driving Force

According to Owen [1] and Vanneste et al. [47], brittle deformation corresponds to cohesive behavior, when increase in pore water pressure is not enough to liquefy sediments. Rossetti and Góes [48] pointed out that presence of these types of faults and their association with undeformed strata corresponds to a brittle deformation when sediments are either unconsolidated or partly consolidated.

## 5. Discussions

### 5.1. Deformation Mechanisms

Soft-sediment deformation structures are formed by disturbances made to nonlithified, water-saturated sedimentary layers [37]. Deformation mechanisms have been investigated by many researchers [1, 17, 35, 37, 38, 44, 49]. If the driving force results in reverse density then slope failure due to liquidization, slumping, or shear stresses may occur [41, 49]. As previously mentioned [1, 37, 39, 45], different driving forces can occur at the same time. Liquidization can be divided into the four types: thixotropy, sensitivity, liquefaction, and fluidization [1]. The origin of soft-sediment deformation structures occur due to these processes. In most cases the triggering mechanism for the deformation mechanisms is considered as an external effect such as artesian flow, groundwater fluctuations, earthquakes, storm currents, and gravity [1, 2, 7, 35]. Deformation mechanisms and driving forces may be different for each of the different structure.

### 5.2. Triggering Mechanisms

There are various possible trigger mechanisms for soft-sediment deformation. The best known are (1) sediment loading [39, 50], (2) storm currents [51–53], and (3) seismicity [5, 7, 12, 14, 16, 35, 54, 55].

Considering (1), the sudden excessive application of load due to irregular and rapid deposition on water-saturated sediments may constitute an affective triggering mechanism [2, 55]. Sediment loading appears to be of minor importance in the Kolankaya Formation, since we are unable to verify such large events of sediment transportation into the basin.

(2) Storm currents can be a triggering mechanism [52], but the deformation structures in the study area show no evidence for formation by such currents. According to Alfaro et al. [53], a minimum 6 m of wave height can produce liquefaction but we consider it unlikely that waves of this height occurred during deposition of the Kolankaya formation, since sedimentation occurred in a restricted-width lake environment.

(3) In this study, seismicity is the likeliest triggering mechanism that could have given rise to the forming soft-sediment deformation structures. This is because the Denizli Basin is a seismically active graben [24, 56] with the large faults that bound the Karakova horst having generated earthquakes in the past and have played important roles in strata tilting during basin development. We relate the soft-sediment deformation structures described from the Kolankaya formation to seismites, based on comparisons shapes and dimensions in the field and experimental literature [2–4, 7, 12–14, 18, 19, 55].

There is a close observed relationship between soft-sediment deformation structures and the earthquake magnitude [7, 9, 12, 57–59]. Some researchers [58] propose that earthquake magnitude should be >4.5 for liquefaction, whilst Scott and Price [9] pointed out that there is no liquefaction observed between 4 and 20 km from an epicenter for earthquake magnitudes lower than 5 and 7, respectively. The distance of faults which are bounding the Denizli Basin varies between 15 and 28 km. It is known that the basin has been subject to large and damaging earthquakes according to historical and recent data [56]. There is also agreement between the observed structures and a classification scale for soft-sediment deformation structures proposed by Rodríguez-Pascua et al. [12]; *M* > 5 for sand dikes and pseudonodule.

**Figure 7:**
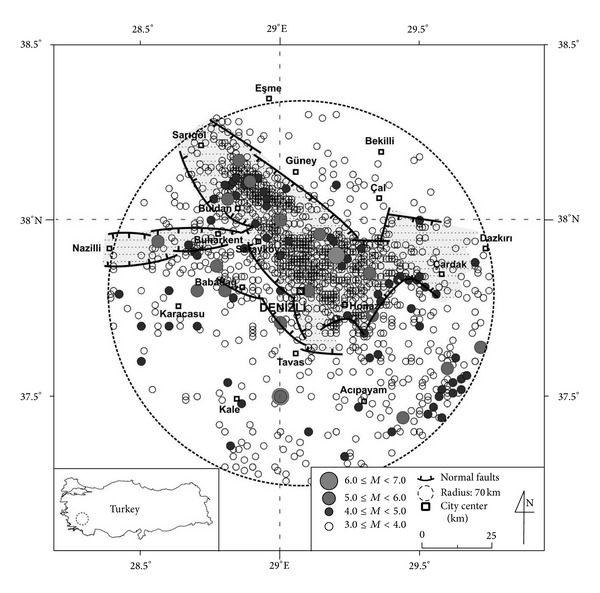
Distribution of the earthquake epicenters (*M* > 3) from A.C. 60 to 2013 in the area of Denizli centered 70 km radius circle (modified from Utku [60], data from Boğaziçi University, Kandilli Observatory and Earthquake Research Institute).

Historical and instrumental earthquake data demonstrate that the area is seismically active (Table 1). Distribution of the earthquake epicenters (*M* > 3) from A.C. 60 to 2013 and relations with the fault systems were illustrated in Figure 7. In historical and instrumental period there are many earthquakes (*M* > 5) in the area of Denizli centered 70 km radius circle (the biggest circular geographic area between 37.26°–38.30°N and 28.39°–29.75°E) [60]. Epicenters are concentrated in the basin and these earthquakes produced by basin and boundary faults.

Papathanassiou et al. [61] proposed a relation between liquefaction occurrence and epicentral distance using earthquakes between 1509 and 2003 in Aegean region and caused liquefactions. The earthquakes (*M*s = 5–7.6) from normal faults which are general fault characteristic of the region, they proposed a *M* = 7 earthquake could cause liquefaction in 60 km range. Castilla and Audemard [62] used a worldwide database to argue that blow diameter versus epicentral distance follows an inverse power law, such that larger-diameter sand volcanoes are restricted to areas proximal to the epicenter, whereas smaller-diameter sand volcanoes occur at much greater distances, up to ~450 km from the seismic source. In this study, deformation structures called seismites located along Karakova uplift which is located in middle parts of the basin and this area is close to boundary faults. Southern bound of Denizli Basin is NW trending Babadağ fault which is consisted of 3 segments and the length of biggest segment is 12 km. SE bound of the basin is E-W trending Honaz fault and divided to 2 segments of 6 km length. The Northern bound of the basin is NW-trending Pamukkale fault which consisted of 4 segments and the biggest segment is 12 km [56]. It is clear that a possible earthquake in basin could produce liquefaction in the Karakova uplift.

## 6. Conclusions

The Denizli Basin is a seismically active graben in the Aegean extensional province, where the Neogene Kolankaya formation is composed of clay, mud, marl, silt, sand, and gravel, deposited in a lacustrine fan delta environment.

We describe for the first time soft-sediment deformation structures in coarse-grained sands, muds, marls, and pebbly sands of the formation.

The deformation mechanisms and driving forces of these structures are compared with those known in the literature: load casts, clastic dikes and sills, disturbed laminae, convolute lamination, slump structures, and synsedimentary faults occurred due to density differences or uneven loading, injection of liquidized sands and pebbly sands, ductile deformation, gravitational instabilities associated with inverse density gradients, gravitational downslope movements, and brittle deformation, respectively.

Regional geological data and field observations indicate that available triggering mechanism for the soft-sediment deformation structures is seismicity due to active extensional normal faulting rather than deformation related to storm activity or sediment loading.

In considering prone to liquidization of sandy lithologies in the Kolankaya Formation and sizes and shapes of structures found, these structures were interpreted as a result of seismic events caused by extension of the Denizli Basin.

## Figures and Tables

**(a):** 

Historical Earthquakes					
Number	Date	Latitude	Longitude	Intensity	Place
1	60	37.9	29.2	IX	Denizli, Pamukkale, Honaz,
2	1703	38	36	VIII	Denizli, Sarayköy, Pamukkale
3	1886	38	29	VI	Denizli
4	1887	38	29	VII	Denizli
5	1899	38	29	VI	Denizli

**(b):** 

Instrumental Earthquakes					
No	Date	Latitude	Longitude	Depth	Magnitude
1	01/01/1904	37.8	29.1	20	4.8
2	08/07/1910	37.8	28.7	30	5.3
3	07/04/1920	37.5	29	15	5.0
4	07/04/1920	37.5	29	15	5.2
5	11/20/1922	37.5	29	28	4.9
6	12/06/1922	37.5	29	15	5.2
7	09/11/1923	38	29.5	22	4.6
8	09/01/1925	37.56	29.17	130	5.4
9	09/03/1925	38	29	15	4.5
10	05/08/1929	38	29.5	15	4.5
11	08/17/1933	37.36	28.82	60	4.5
12	08/12/1936	37.44	29.44	130	5.0
13	12/21/1945	37.9	29	4	4.7
14	01/13/1948	38.1	28.8	30	4.8
15	12/19/1958	37.81	29.52	40	4.5
16	06/21/1961	37.87	28.77	60	5.0
17	03/11/1963	37.96	29.14	40	5.5
18	06/13/1965	37.85	29.32	33	5.7
19	06/17/1965	37.6	28.8	33	4.6
20	06/17/1965	37.77	29.36	37	4.5
21	07/12/1965	37.62	29.35	50	4.4
22	07/12/1965	37.62	29.35	50	4.5
23	10/04/1965	37.75	29	10	4.2
24	12/02/1965	37.61	29.32	38	4.6
25	08/16/1966	37.47	29.28	79	4.2
26	07/19/1967	38.1	28.87	41	4.9
27	07/25/1967	37.8	28.6	75	4.2
28	07/25/1967	37.9	28.7	101	4.5
29	11/13/1967	37.78	28.83	34	4.5
30	03/28/1969	38.09	29.02	29	4.5
31	03/28/1970	38.1	29.2	33	4.7
32	02/20/1971	37.82	29.39	36	4.5
33	05/12/1971	37.58	29.6	33	5.2
34	05/12/1971	37.5	29.57	49	4.2
35	05/14/1971	37.47	29.55	8	4.6
37	10/30/1973	37.37	29.05	19	4.0
39	08/15/1976	37.84	28.77	11	5.4
40	08/19/1976	37.71	29	20	5.1
41	09/10/1977	37.99	28.75	10	4.0
42	01/11/1978	37.48	28.86	5	5.0
43	06/17/1978	37.54	28.81	10	4.1
47	01/09/1982	37.92	28.87	3	4.4
48	11/23/1982	37.45	29.53	17	4.3
49	06/24/1983	37.84	29.5	10	4.2
51	12/09/1983	37.83	29.42	6	4.2
52	03/25/1984	37.7	28.7	10	4.3
55	10/11/1986	37.94	28.56	5	5.4
57	02/24/1989	37.73	29.33	10	4.8
58	02/24/1989	37.72	29.26	19	4.2
59	02/24/1989	37.73	29.24	23	4.3
64	08/18/1995	37.78	29.47	18	4.5
66	01/21/1997	38.08	29	18	5.0
68	02/25/1998	37.78	29.62	23	4.0
69	04/21/2000	37.88	29.36	20	4.8
71	10/04/2000	37.9	29.03	3	4.6
73	07/30/2002	37.73	29.22	6	4.2
74	07/23/2003	38.14	28.86	4	5.0
75	07/26/2003	38.11	28.87	6	4.7
76	07/26/2003	38.11	28.89	7	5.3
77	07/26/2003	38.14	28.85	5	4.4
78	02/20/2013	37.91	29.32	22.78	4.0
79	02/05/2011	37.90	29.05	10.09	4.0
80	12/04/2009	37.41	29.56	25.02	4.8
81	12/04/2009	37.92	28.84	13.94	4.8
82	11/25/2009	37.92	28.86	12.15	4.5
83	03/22/2009	37.92	29.11	9.42	4.0
84	12/24/2008	37.89	29.23	9.91	4.0
85	04/25/2008	37.83	29.26	18.83	5.0
86	01/10/2008	37.92	28.79	17.31	4.1
